# Pulmonary artery systolic pressure associated with inflammatory factors among pediatric congenital heart disease with pulmonary arterial hypertension after cardiopulmonary bypass

**DOI:** 10.1016/j.jped.2025.01.006

**Published:** 2025-02-27

**Authors:** WenJuan Li, Wenyuan Shang, Jihong Huang

**Affiliations:** Xinhua Hospital, Affiliated to Shanghai Jiao Tong University School of Medicine, Department of Pediatric Cardiology, Shanghai, China

**Keywords:** Inflammatory factors, Pulmonary arterial hypertension secondary to congenital heart disease (PAH-CHD), Cardiopulmonary bypass

## Abstract

**Objective:**

This study aimed to evaluate the perioperative inflammatory cytokines in pediatric patients with pulmonary arterial hypertension secondary to congenital heart disease and also sought to investigate the correlation between preoperative echocardiographic pulmonary artery systolic pressure and inflammatory factors after cardiopulmonary bypass in these patients.

**Methods:**

A retrospective observational study was conducted involving 59 children under 2 years old. Echocardiography was used to measure tricuspid annular peak systolic velocity. The levels of perioperative inflammatory cytokines in the plasma, including IL-6, IL-8, IL-10, IL-1β, and TNF-α, were measured. Additionally, postoperative ventilation time, length of intensive care unit stay, and ward stay were recorded.

**Results:**

Patients with pulmonary hypertension experience longer postoperative ventilation time, intensive care unit stay, and ward stay. Although no significant differences were observed in the cardiopulmonary bypass and aortic cross-clamping, there was a more pronounced increase in postoperative inflammatory cytokines, including IL-6, IL-8, and IL-10, in these patients following cardiopulmonary bypass (*p* < 0.05). Preoperative echocardiographic pulmonary artery systolic pressure was found to be associated with the levels of IL-6 and IL-10 after surgery in pulmonary hypertension patients. A pulmonary artery systolic pressure greater than 52 mmHg was able to predict a postoperative ventilation time exceeding 21 h.

**Conclusion:**

Higher levels of inflammatory cytokines were observed in pediatric patients with pulmonary hypertension secondary to congenital heart disease following cardiopulmonary bypass. Additionally, preoperative elevation in echocardiographic pulmonary artery systolic pressure was associated with increased postoperative inflammatory markers, suggesting a potential correlation with adverse early postoperative clinical outcomes.

## Introduction

Tremendous progress has occurred in the field of treating children with congenital heart disease (CHD) over the past decades, making the ongoing quest for improving outcomes crucial for children with CHD after surgery. There are several perioperative factors that determine outcomes, including the type of CHD, risk category, age, gender, duration of cardiopulmonary bypass (CPB), and aortic cross-clamp (ACC) time.^1^ PAH is also one of the most important causes of postoperative morbidity and mortality in children after cardiac surgery.^2^^,^^3^ PAH exhibits an estimated annual incidence and point prevalence averaging 2.2 and 15.6 cases per million children.^4^ PAH secondary to CHD (PAH—CHD) is a major subtype of PAH, defined as a mean pulmonary arterial pressure (mPAP) > 20 mmHg.^5^^,^^6^ Left-to-right shunts CHD, including ventricular septal defects (VSD) and atrial septal defects (ASD), are the most common types of CHD. These conditions are often complicated by pulmonary arterial hypertension (PAH) of varying severity due to increased pulmonary blood flow.^2^

Although early surgical repair can block this abnormal shunt and reverse pulmonary hypertension, patients with corrected PAH—CHD seem to have a poor prognosis compared with other types of CHD.^7^^,^^8^ The cause is largely due to the closure of a large left-to-right shunt that triggers a series of events in the vascular function of the pulmonary arteries, leading to ventricular dysfunction.^9^^,^^10^ It is also partly related to myocardial and pulmonary dysfunction after cardiopulmonary bypass (CPB). CPB is associated with systemic inflammatory response syndrome (SIRS) and induces a variety of cytokines that may be linked to postoperative complications.

The concept that cytokines, chemokines, and perivascular inflammatory cell infiltrates play a role in PAH has gained support. Elevated circulating levels of inflammatory cytokines and increased pulmonary expression of several chemokines have been observed in adult patients with PAH.^11^ Studies revealed that PAH exacerbates the production of inflammatory cytokines compared to patients without PAH by inducing a systemic inflammatory response through the activation of complement and leukocytes and the expression of adhesive cytokines.^12^^,^^13^

However, there are few reports on perioperative inflammatory cytokines in pediatric patients with PAH—CHD after CPB. It is unknown whether preoperative PAH contributes to a stronger systemic inflammatory response after CPB in these patients and then leads to poor outcomes. In this study, the authors evaluated the plasma levels of inflammatory cytokines before and after CPB. The authors also investigated the association between PAH and postoperative inflammatory cytokines in pediatric PAH—CHD patients.

## Material and methods

### Study design

This retrospective observational study was conducted in the Department of Pediatric Cardiology between August 2021 and March 2022. Fifty-nine children (32 males and 27 females) were analyzed. This work was approved by the Hospital Ethics Committee. All procedures were followed in accordance with the ethical standards of the committee responsible for human experimentation (institutional or regional) and with the Helsinki Declaration of 1975. Informed consent was not required in the present study. The study cohort consisted of two groups. The PAH—CHD group included 34 patients, and the No PAH—CHD group consisted of 25 patients. Study flow chat was shown in Supplement Figure 1. Inclusion criteria included children under 2 years old diagnosed with unrestricted left-to-right shunts CHD by echocardiography. Exclusion criteria included neonates, individuals with acute infections, arrhythmia, respiratory distress, recent administration of vasoactive drugs within 1 week before surgery, patients in the ICU before surgery, and those with a residual shunt after surgery.

**Figure 1:**
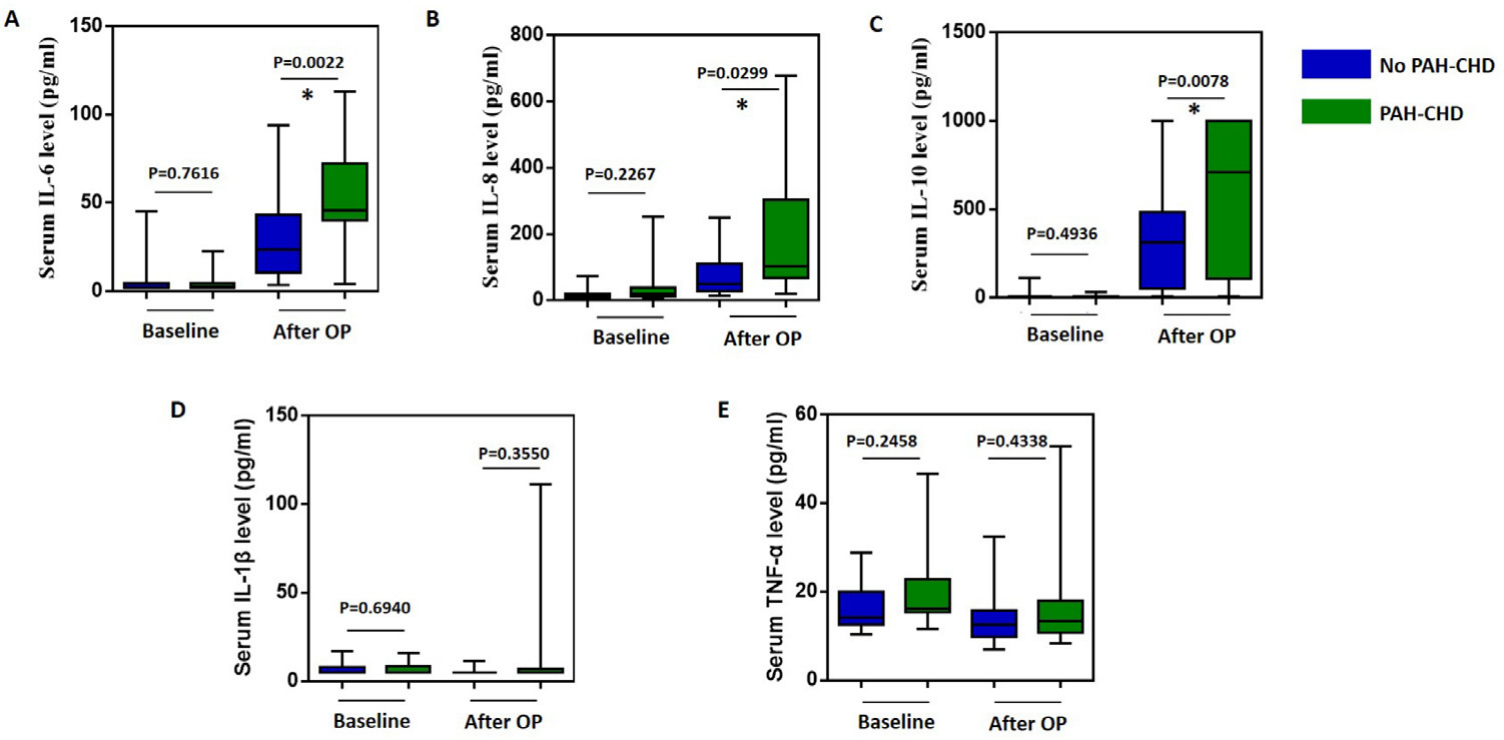
Perioperative inflammatory factors for pediatric CHD patients with and without PAH.

Operation data, including aortic cross-clamping time and cardiopulmonary bypass time, were recorded. Early postoperative outcomes, such as duration of mechanical ventilation and length of stay in the ICU and ward after surgery, were also assessed. Peripheral venous blood samples were taken preoperatively and 2 h after CPB. Samples were collected in tubes containing potassium ethylenediaminetetraacetic acid. According to the manufacturer's procedure, the levels of IL-1β, TNF-α, IL-6, IL-8, and IL-10 were determined by enzyme-linked immunosorbent assay.

Transthoracic Doppler echocardiography is considered the primary screening tool for detecting PAH due to the strong correlation between estimated right ventricular systolic pressure and invasively measured pulmonary artery pressure.^14^ In this study, echocardiography evaluations were conducted under post-induction sedation. Continuous-wave Doppler echocardiographic sampling of TRV was performed using the modified Bernoulli equation (4 [TRV] 2).^15^ The estimation of right ventricular systolic pressure (RVSP) by Doppler transthoracic echocardiography (TTE) assessment of tricuspid valve regurgitation (TR) jet peak velocity accurately predicts the PASP observed by invasive measurement. A TR velocity > 2.8 m/*sec* is considered a reasonable cutoff to define elevated pulmonary pressures in the absence of pulmonary stenosis of PAH—CHD patients.^16^^,^^17^

### Patients’ management

All patients had hemodynamic stability before surgery, and none of them had Eisenmenger syndrome. The anesthetic management and monitoring were performed as follows: Induction of anesthesia included midazolam and fentanyl. After the rocuronium administration, the trachea of the patients was intubated. Standard monitoring was performed, including an arterial line for continuous blood pressure monitoring and blood gas analysis, and a double-lumen central venous catheter was inserted to measure central venous pressure, deliver vasoactive drugs, and repeatedly monitor venous oxygen saturations. Anesthesia was maintained with propofol and additional fentanyl. Vasopressors were administered at the discretion of the attending anesthesiologist. Cardiac surgical procedures were performed under routine general anesthesia and CPB, and all patients had no residual shunt and PAH after surgery. Midazolam and dexmedetomidine were used as sedatives upon arrival in the pediatric cardiac ICU (PCICU), none of the patients were extubated in the operating room, and daily spontaneous breathing trial (DSBT) protocol^18^ was used to assess patients’ readiness for extubation when the trachea will be extubated in the PCICU. If all screening criteria were met, patients were placed on continuous positive airway pressure of 5 cm H2O with pressure support of 8 cm H_2_O for up to 2 h. Extubations were performed if this was well tolerated, and supplemental oxygen was delivered via nasal cannula, following approval from the intensivist approval.

### Statistical analysis

Data were analyzed using SPSS 18.0. Data were expressed as either mean value ± standard deviation or median and interquartile ranges for continuous variables and as percentages or numbers for categorical variables. The Student's *t*-test was used for analyzing normally distributed numeric variables, the Mann-Whitney U test was used for non-normally distributed numeric variables, and the chi-square test was used for analyzing categorical variables. The correlation of each variable with clinical outcomes was evaluated using the Spearman test. A 2-sided *p*‐value of < 0.05 indicated statistical significance.

## Results

### Patient characteristics and clinical early postoperative outcomes

During the study period, 59 children under 2 years old were referred to the surgical unit for congenital heart disease with a left-to-right shunt; 27 patients were female (45.76%), and 32 patients were male (54.24%). Echocardiography assessed patients before cardiac surgery; 34 patients (57.63%) had PAH, and 25 patients (42.37%) did not have PAH. Preoperative PASP was 59.94 mmHg ± 17.56 mmHg in the PAH group. All patients underwent successful closure of the cardiac shunt. Demographic data and associated congenital heart lesions are presented in Table 1. There were no significant differences in the demographic characteristics of the patients between those with PAH and those without PAH. No significant difference in the preoperative serum levels of inflammatory cytokines, including IL-8, IL-6, IL-10, IL-1β, and TNF-α, was found between these two groups (Table 1).

**Table 1:** Demographic data and outcomes of patients with and without PAH.

Variable	No PAH group (*n* = 25)	PAH group (*n* = 34)	*p*-value
Age (months)	7.90 ± 0.75	6.62 ± 1.06	0.3597
Gender			*0.8173*
Male (n)	14	18	
Female (n)	11	16	
Weight (Kg)	7.49 ± 0.36	6.60 ± 0.39	*0.1162*
PASP (mmHg)	< 20 mmHg	56.73±20.58	<0.0001
Type of CHD			*0.1693*
VSD (n)	10	17	
PAPVC (n)	8	3	
PDA/VSD (n)	1	1	
VSD/ASD (n)	4	7	
VSD/MR (n)	2	1	
DORV/VSD (n)	0	2	
COA/VSD (n)	0	3	
VIS	11.36 ± 0.44	11.76 ± 0.41	*0.5089*
Pre-IL-6 (pg/ml)	6.45 ± 2.79	5.52 ± 1.52	*0.7616*
Pre-IL-8 (pg/ml)	20.58 ± 5.54	44.10 ± 16.62	*0.2267*
Pre-IL-10 (pg/ml)	12.31 ± 6.79	7.98 ± 1.43	*0.4936*
Pre-IL-1β (pg/ml)	6.70 ± 0.80	7.18 ± 0.90	*0.6940*
Pre-TNF-α (pg/ml)	16.47 ± 1.44	19.51 ± 1.98	*0.2458*
Mechanical ventilation time (hours)	13.72 ± 2.51	22.76 ± 2.49	*0.0485*
ICU stay	3.33± 0.34	4.36 ± 0.34	*0.0432*
Ward stay	8.56 ± 0.49	11.61 ± 0.78	*0.0039*

PAH, pulmonary atrial hypertension; PASP, pulmonary artery systolic pressure; VSD, ventricular septal defect; PAPVC, Partial anomalous pulmonary venous connection; PDA, Patent ductus arteriosus; CAVC, Complete atrioventricular septal defect; COA, Coarctation of aorta; VIS, vasoactive-inotropic score.

There was no mortality in either group. The PAH group had a significant impact on early postoperative surgical outcomes; the duration of mechanical ventilation time was significantly longer in the PAH group (22.76 h ± 2.49 h) compared with the No PAH group (13.72 h ± 2.51 h) (*p* = 0.0485). Importantly, the lengths of ICU and ward stay after surgery were also longer in the PAH group than in the No PAH group (*p* = 0.0432 and 0.0039, respectively).

### Change of inflammatory cytokines after CPB

After surgery, CPB can produce and release numerous pro-inflammatory cytokines. The authors analyzed the changes in postoperative inflammatory cytokines. As shown in Figure 1, the levels of inflammatory cytokines, including IL-6, IL-8, and IL-10, increased significantly after CPB in both PAH and non-PAH patients (*p* < 0.05). However, the levels of IL-1β and TNF-α did not show significant changes. Interestingly, these inflammatory cytokines increased more in the PAH group compared to the non-PAH group (*P* = 0.0380, 0.0359, and 0.0078, respectively). Then the authors compared the duration of CPB time and aortic cross-clamping (ACC) time of these two groups. The CPB time for patients with PAH and without PAH was (66.64 ± 7.53) h and (75.63 ± 3.65) h, respectively. The ACC time was (48.19 ± 6.23) min for PAH patients and (53.22 ± 3.32) min for patients without PAH. No significant difference was found in the CPB and ACC durations between PAH and non-PAH patients (*p* = 0.2438 and 0.4510).

To investigate whether preoperative PAH is associated with higher levels of inflammatory cytokines, Spearman correlation coefficients were calculated between preoperative PASP and postoperative levels of inflammatory cytokines in the PAH—CHD group. As shown in Table 2, preoperative PASP was found to be correlated with postoperative levels of IL-6 (rs = 0.5156, *p* = 0.0083) and IL-10 (rs = 0.5027, *p* = 0.0397), but not with the level of IL-8.

**Table 2:** Spearman's correlation coefficient analysis between PASP and postoperative inflammatory cytokines.

Spearman's correlation coefficient						
	Post-IL 6		Post-IL 8		Post-IL 10	
	*r_s_*	*p*-value	*r_s_*	*p*-value	*r_s_*	*p*-value
Pre- PASP	0.5156	*0.0083*	0.1099	*0.6263*	0.5027	*0.0397*

### Preoperative PASP is associated with prolonged postoperative mechanical ventilation

**Figure 2:**
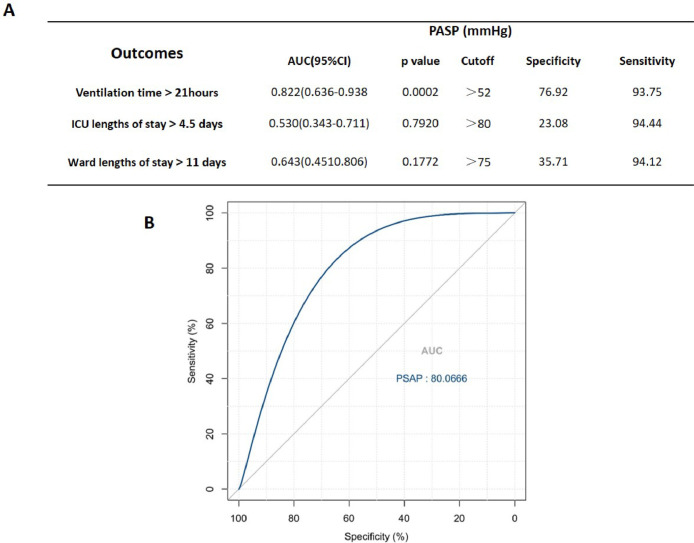
Preoperative PASP predict prolonged mechanical ventilation. A, Preoperative PASP for early postoperative outcomes after CPB. B, ROC curve for preoperative PASP for predicting postoperative ventilation time >21 h.

CPB causes a series of inflammatory events that have adverse effects on postoperative outcomes, and the release of cytokines plays a key role in the pathophysiology of this process. Given that preoperative high PASP is associated with higher levels of IL-6 and IL-10 after CPB, the authors hypothesize that preoperative high PASP, linked to more robust inflammatory responses by CPB, leads to subsequent poor postoperative outcomes in pediatric patients with PAH—CHD. To verify this hypothesis, the study assessed the reliability of preoperative PASP in predicting early postoperative outcomes. The results summarized in Figure 2 show the sensitivity, specificity, AUC, and p-value of preoperative PASP for predicting ventilation time > 21 h, ICU length of stay > 4.5 days, and ward length of stay > 11 days. Preoperative PASP > 52 mmHg is associated with ventilation time > 21 h, but not with ICU length of stay > 4.5 days and ward length of stay > 11 days.

## Discussion

PAH is one of the major challenges in pediatric surgery for congenital heart disease, which is a crucial factor affecting morbidity and mortality in the early postoperative follow-up.^9^^,^^19^ PAH associated with left-to-right shunts caused by high pulmonary arterial flow can be reversed by early closure of the shunt before a progressive increase in pulmonary vascular resistance (PVR) and subsequent remodeling of the pulmonary vasculature,^20^^,^^21^ however, CHD patients with preoperative PAH seem to have an unfavorable prognosis compared with other non-PAH. Therefore, it is crucial to continue searching for ways to improve outcomes for PAH—CHD patients. The findings of the present study indicate that inflammatory cytokines, including IL-6, IL-8, and IL-10, rise after CPB in both PAH—CHD and non-PAH CHD patients. The levels of these inflammatory cytokines were significantly higher in PAH—CHD patients. Based on the Spearman correlation coefficient analysis, a higher preoperative PASP, used to assess the severity of PAH, is associated with the levels of IL-6 and IL-10 after CPB. A preoperative PASP greater than 52 mmHg could predict ventilation time exceeding 21 h after surgery, as determined by ROC analysis.

Inflammation is a prominent feature in the development of PAH, and elevated circulating levels of cytokines have been reported in patients with PAH, such as TNF-α, IL-1β, and IL-6.^22^, ^23^, ^24^ However, the authors did not detect increasing levels of TNF-α, IL-1β, and IL-6 in the preoperative PAH—CHD patients. The high pulmonary hypertension observed in this study may be attributed to increased pulmonary blood flow, rather than the remodeling of small to medium-sized pulmonary arteries, which is characterized by increased endothelial cell activation and platelet-mediated inflammation.^25^ CPB is generally accompanied by a systemic inflammatory response that increases the levels of cytokines and acute phase reactants such as CRP, IL-6, IL-8, IL-10, IL-1β, and TNF-α, due to surgical trauma, blood contact with the surface of the extracorporeal circulation devices, blood loss, and ischemic reperfusion injury. These inflammatory cytokines, such as IL-6, IL-8, IL-1β, and TNF-α, subsequently elevate the level of activated neutrophils, leading to cellular damage, increased leukocyte activation, chemotaxis, and leukocyte-endothelial adherence.^12^^,^^26^ In the present study, IL-6, IL-8, and IL-10 significantly increased after CPB in both groups. However, TNF-α and IL-1β remained unchanged compared to preoperative levels in both groups. This could be attributed to the fact that TNF-α and IL-1β levels rise early after cardiac surgery, while IL-6 and IL-8 peak at a later stage.^27^ In the present results, although no difference was found between patients with PAH and those without PAH during CPB time, levels of IL-6, IL-8, and IL-10 increased significantly in PAH patients after CPB.

Previous studies have revealed that cardiac surgery involving CPB is associated with less favorable outcomes, as it activates an inflammatory response that releases cytokines.^28^^,^^29^ Holmes IV et al. demonstrated that patients with an exaggerated inflammatory response to CPB tend to require more respiratory support. Therefore, the magnitude of the inflammatory response to CPB adversely influences clinical outcomes.^30^ This study confirmed that patients with PAH—CHD had higher levels of inflammatory cytokines after CPB, which resulted in longer postoperative ventilation time, ICU stay, and ward stay compared to non-PAH patients. Additionally, preoperative PASP was associated with longer postoperative ventilation time. Although patients with PAH had longer ICU and general ward after surgery, this study did not find any correlation with preoperative PASP based on ROC analysis.

There are still limitations to this study. First, the SPAP of patients with CHD-PAH was measured using echocardiography, which was not very close compared to measurements obtained through cardiac catheterization. Secondly, this is a retrospective study with a small sample size, and further prospective large-scale studies are needed.

## Conclusion

Pediatric patients with PAH—CHD exhibited a higher systemic inflammatory response after CPB. Preoperative echocardiographic PASP is correlated with the levels of IL-6 and IL-10, which may contribute to unfavorable early postoperative clinical outcomes.

## Abbreviations

ACC, aortic cross-clamp; ASD, atrial septal defects; CHD, congenital heart disease; CPB cardiopulmonary bypass; ICU, intensive care unit; mPAP, mean pulmonary arterial pressure; PASP, pulmonary artery systolic pressure; PAH, pulmonary arterial hypertension; RVSP, right ventricular systolic pressure; SIRS, systemic inflammatory response syndrome; TRV, tricuspid annular peak systolic velocity; TTE, transthoracic echocardiography; TR, tricuspid valve regurgitation; VSD, ventricular septal defects.

## Funding

This work was supported by the Fundamental Research Funds for the Central Universities (grant number YG2023QNB13) and Xinhua Hospital & Shanghai Jiao Tong University Joint Project (grant number 21XJMR04).

## Author contributions

JHH conceived and designed the study. WJL and WYS collected the data and conducted the statistical analysis. WJL drafted the manuscript. JHH reviewed and edited the manuscript. All authors read and approved the manuscript.

## Ethics approval

This work was approved by the Xinhua Hospital Ethics Committee. All procedures were followed in accordance with the ethical standards of the committee responsible for human experimentation (institutional or regional) and with the Helsinki Declaration of 1975. Informed consent was not required in the present study.

## Availability of data and materials

All data analyzed during this study are included in this article. Further inquiries are available from the corresponding author on reasonable request.

## Conflicts of interest

The authors declare no conflicts of interest.

## Appendix Supplementary materials

Image, application 1

## References

[1] Mehmood A., Nadeem R.N., Kabbani M.S., Khan A.H., Hijazi O., Ismail S.R. (2019). Impact of cardiopulmonary bypass and aorta cross clamp time on the length of mechanical ventilation after cardiac surgery among children: a Saudi Arabian Experience. Cureus.

[2] Bando K., Turrentine M.W., Sharp T.G., Sekine Y., Aufiero T.X., Sun K. (1996). Pulmonary hypertension after operations for congenital heart disease: analysis of risk factors and management. J Thorac Cardiovasc Surg.

[3] Galiè N., Hoeper M.M., Humbert M., Torbicki A., Vachiery J.L., Barbera J.A. (2009). Guidelines for the diagnosis and treatment of pulmonary hypertension: the Task Force for the Diagnosis and Treatment of Pulmonary Hypertension of the European Society of Cardiology (ESC) and the European Respiratory Society (ERS), endorsed by the International Society of Heart and Lung Transplantation (ISHLT) [Erratum in: Eur Heart J. 2011;32:926]. Eur Heart J..

[4] van Loon R.L., Roofthooft M.T., Hillege H.L., ten Harkel A.D., van Osch-Gevers M., Delhaas T. (2011). Pediatric pulmonary hypertension in The Netherlands: epidemiology and characterization during the period 1991 to 2005. Circulation.

[5] Dimopoulos K., Wort S.J., Gatzoulis M.A. (2014). Pulmonary hypertension related to congenital heart disease: a call for action. Eur Heart J.

[6] Duffels M.G., Engelfriet P.M., Berger R.M., van Loon R.L., Hoendermis E., Vriend J.W. (2007). Pulmonary arterial hypertension in congenital heart disease: an epidemiologic perspective from a Dutch registry. Int J Cardiol.

[7] Alonso-Gonzalez R., Lopez-Guarch C.J., Subirana-Domenech M.T., Ruíz J.M., González I.O., Cubero J.S. (2015). Pulmonary hypertension and congenital heart disease: an insight from the REHAP National Registry. Int J Cardiol.

[8] Manes A., Palazzini M., Leci E., Bacchi Reggiani M.L., Branzi A., Galiè N. (2014). Current era survival of patients with pulmonary arterial hypertension associated with congenital heart disease: a comparison between clinical subgroups. Eur Heart J.

[9] Schulze-Neick I., Li J., Penny D.J., Redington A.N. (2001). Pulmonary vascular resistance after cardiopulmonary bypass in infants: effect on postoperative recovery. J Thorac Cardiovasc Surg.

[10] Kaestner M., Schranz D., Warnecke G., Apitz C., Hansmann G., Miera O. (2016). Pulmonary hypertension in the intensive care unit. Expert consensus statement on the diagnosis and treatment of paediatric pulmonary hypertension. The European Paediatric Pulmonary Vascular Disease Network, endorsed by ISHLT and DGPK. Heart.

[11] Dorfmüller P., Perros F., Balabanian K., Humbert M. (2003). Inflammation in pulmonary arterial hypertension. Eur Respir J.

[12] Soares L.C., Ribas D., Spring R., Silva J.M., Miyague N.I. (2010). Perfil clínico da resposta inflamatória sistêmica após cirurgia cardíaca pediátrica com circulação extracorpórea [Clinical profile of systemic inflammatory response after pediatric cardiac surgery with cardiopulmonary bypass]. Arq Bras Cardiol.

[13] Yin X., Xin M., Ding S., Gao F., Wu F., Wang J. (2021). Predictive role of perioperative neutrophil to lymphocyte ratio in pediatric congenital heart disease associated with pulmonary arterial hypertension. BMC Surg.

[14] Budev M.M., Arroliga A.C., Jennings C.A. (2003). Diagnosis and evaluation of pulmonary hypertension. Cleve Clin J Med.

[15] Rudski L.G., Lai W.W., Afilalo J., Hua L., Handschumacher M.D., Chandrasekaran K. (2010). Guidelines for the echocardiographic assessment of the right heart in adults: a report from the American Society of Echocardiography endorsed by the European Association of Echocardiography, a registered branch of the European Society of Cardiology, and the Canadian Society of Echocardiography. J Am Soc Echocardiogr.

[16] McQuillan B.M., Picard M.H., Leavitt M., Weyman A.E. (2001). Clinical correlates and reference intervals for pulmonary artery systolic pressure among echocardiographically normal subjects. Circulation.

[17] Koestenberger M., Nagel B., Ravekes W., Avian A., Heinzl B., Fandl A. (2012). Tricuspid annular peak systolic velocity (S') in children and young adults with pulmonary artery hypertension secondary to congenital heart diseases, and in those with repaired tetralogy of Fallot: echocardiography and MRI data. J Am Soc Echocardiogr.

[18] Abu-Sultaneh S., Hole A.J., Tori A.J., Benneyworth B.D., Lutfi R., Mastropietro C.W. (2017). An Interprofessional Quality Improvement Initiative to Standardize Pediatric Extubation Readiness Assessment. Pediatr Crit Care Med.

[19] Wheller J., George B.L., Mulder D.G., Jarmakani J.M. (1979). Diagnosis and management of postoperative pulmonary hypertensive crisis. Circulation.

[20] van der Feen D.E., Bartelds B., de Boer R.A., Berger R.M. (2019). Assessment of reversibility in pulmonary arterial hypertension and congenital heart disease. Heart.

[21] McLaughlin V.V., Gaine S.P., Howard L.S., Leuchte H.H., Mathier M.A., Mehta S. (2013). Treatment goals of pulmonary hypertension. J Am Coll Cardiol.

[22] Soon E., Holmes A.M., Treacy C.M., Doughty N.J., Southgate L., Machado R.D. (2010). Elevated levels of inflammatory cytokines predict survival in idiopathic and familial pulmonary arterial hypertension. Circulation.

[23] Humbert M., Monti G., Brenot F., Sitbon O., Portier A., Grangeot-Keros L. (1995). Increased interleukin-1 and interleukin-6 serum concentrations in severe primary pulmonary hypertension. Am J Respir Crit Care Med.

[24] Tuder R.M., Voelkel N.F. (1998). Pulmonary hypertension and inflammation. J Lab Clin Med.

[25] Brun H., Holmstrøm H., Thaulow E., Damås J.K., Yndestad A., Aukrust P. (2009). Patients with pulmonary hypertension related to congenital systemic-to-pulmonary shunts are characterized by inflammation involving endothelial cell activation and platelet-mediated inflammation. Congenit Heart Dis.

[26] Laffey J.G., Boylan J.F., Cheng D.C. (2002). The systemic inflammatory response to cardiac surgery: implications for the anesthesiologist. Anesthesiology.

[27] Wheller J., George B.L., Mulder D.G., Jarmakani J.M. (1979). Diagnosis and management of postoperative pulmonary hypertensive crisis. Circulation.

[28] Hirai S. (2003). Systemic inflammatory response syndrome after cardiac surgery under cardiopulmonary bypass. Ann Thorac Cardiovasc Surg.

[29] Nebelsiek T., Beiras-Fernandez A., Kilger E., Möhnle P., Weis F. (2012). Routine use of corticosteroids to prevent inflammation response in cardiac surgery. Recent Pat Cardiovasc Drug Discov.

[30] Holmes J.H., Connolly N.C., Paull D.L., Hill M.E., Guyton S.W., Ziegler S.F. (2002). Magnitude of the inflammatory response to cardiopulmonary bypass and its relation to adverse clinical outcomes. Inflamm Res.

