# Supplementary figures and images for: Pulmonary artery systolic pressure associated with inflammatory factors among pediatric congenital heart disease with pulmonary arterial hypertension after cardiopulmonary bypass
Source: J Pediatr (Rio J). 2025 Feb 27;101(3):438–44. doi: 10.1016/j.jped.2025.01.006 (PMC12039507; doi:10.1016/j.jped.2025.01.006)

**JPED-D-24-00321_Supplementary Material**

**Supplement data**


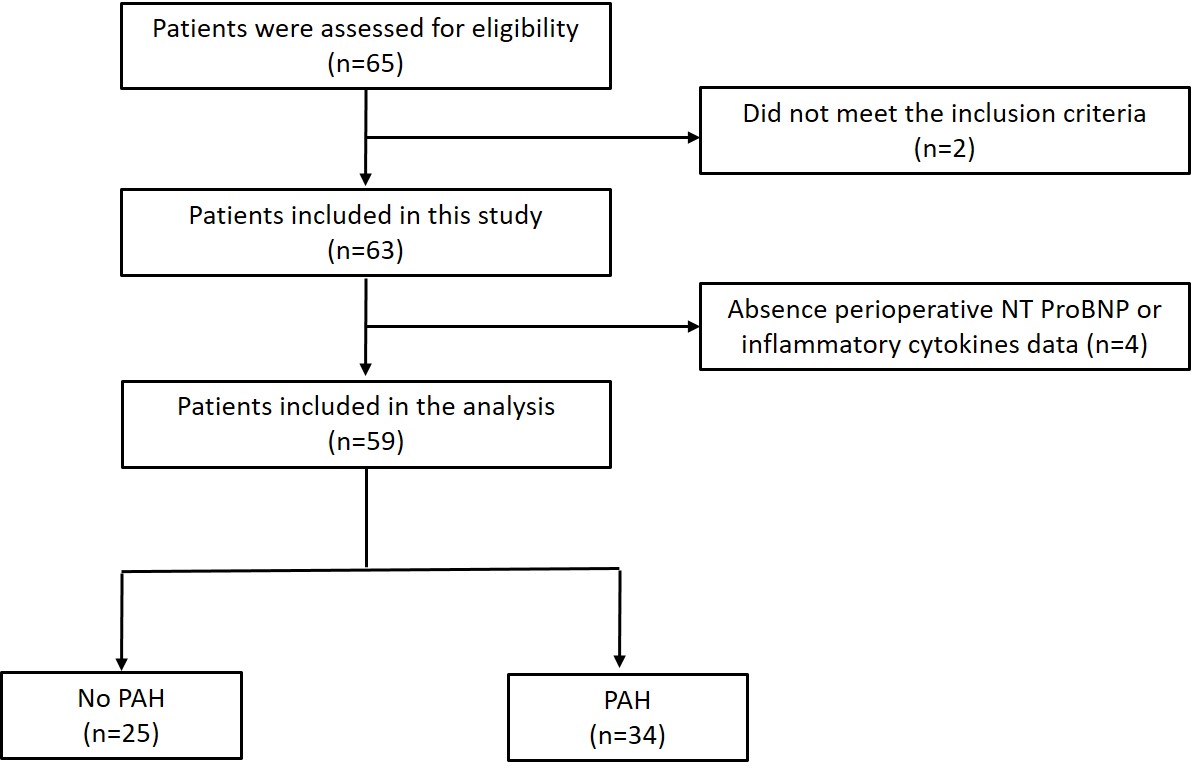


**Supplement Figure 1 Study flow chat**

Supplement: Supplementary file 1 [file mmc1.doc]
